# Injury-experienced satellite cells retain long-term enhanced regenerative capacity

**DOI:** 10.1186/s13287-023-03492-4

**Published:** 2023-09-12

**Authors:** Jacopo Morroni, Anna Benedetti, Lorenza Esposito, Marco De Bardi, Giovanna Borsellino, Carles Sanchez Riera, Lorenzo Giordani, Marina Bouche, Biliana Lozanoska-Ochser

**Affiliations:** 1 Department of Anatomical, Histological, Forensic and Orthopedic Sciences, Section of Histology and Embryology, Sapienza University of Rome, Rome, Italy; 2 Sorbonne Université, INSERM UMRS 974, Association Institut de Myologie, Centre de Recherche en Myologie, 75013 Paris, France; 3 Department of Medicine and Surgery, LUM University, Casamassima, Bari, Italy; 4 Neuroimmunology Unit, IRCCS Santa Lucia Foundation, Rome, Italy; 5 Present Address: COU of Neurology, Fondazione Policlinico Universitario A. Gemelli IRCCS, Rome, Italy; 6 Present Address: Translational Oncology Research Unit, IRCCS Regina Elena National Cancer Institute, Rome, Italy

**Keywords:** Satellite cells, Inflammatory memory, Repeated injury, Muscle regeneration

## Abstract

**Background:**

Inflammatory memory or trained immunity is a recently described process in immune and non-immune tissue resident cells, whereby previous exposure to inflammation mediators leads to a faster and stronger responses upon secondary challenge. Whether previous muscle injury is associated with altered responses to subsequent injury by satellite cells (SCs), the muscle stem cells, is not known.

**Methods:**

We used a mouse model of repeated muscle injury, in which intramuscular cardiotoxin (CTX) injections were administered 50 days apart in order to allow for full recovery of the injured muscle before the second injury. The effect of prior injury on the phenotype, proliferation and regenerative potential of satellite cells following a second injury was examined in vitro and in vivo by immunohistochemistry, RT-qPCR and histological analysis.

**Results:**

We show that SCs isolated from muscle at 50 days post-injury (injury-experienced SCs (ieSCs)) enter the cell cycle faster and form bigger myotubes when cultured in vitro, compared to control SCs isolated from uninjured contralateral muscle. Injury-experienced SCs were characterized by the activation of the mTORC 1 signaling pathway, suggesting they are poised to activate sooner following a second injury. Consequently, upon second injury, SCs accumulate in greater numbers in muscle at 3 and 10 days after injury. These changes in SC phenotype and behavior were associated with accelerated muscle regeneration, as evidenced by an earlier appearance of bigger fibers and increased number of myonuclei per fiber at day 10 after the second injury.

**Conclusions:**

Overall, we show that skeletal muscle injury has a lasting effect on SC function priming them to respond faster to a subsequent injury. The ieSCs have long-term enhanced regenerative properties that contribute to accelerated regeneration following a secondary challenge.

**Supplementary Information:**

The online version contains supplementary material available at 10.1186/s13287-023-03492-4.

## Background

The response of skeletal muscle to acute injury is a highly coordinated process involving recruited and resident immune cells, which together with non-immune muscle cells orchestrate the process of regeneration [1, 2]. Following acute muscle injury, satellite cells (SCs), the muscle stem cells, become activated, proliferate and differentiate into myoblasts, which fuse to form new myofibers [3–5]. This process is facilitated and fine-tuned by pro-inflammatory and pro-regenerative cytokines released by recruited innate immune cells, as well as resident non-immune muscle cells such as fibro-adipogenic progenitors (FAPs) [6–10]. One of the most widely studied models of acute muscle injury involves an intramuscular injection of cardiotoxin (CTX), which leads to clearly defined stages of muscle repair. Neutrophils and inflammatory monocytes are recruited within the first 24 h after CTX injury and are critical for the clearance of dead cells, and the production of inflammatory cytokines is necessary to kick-start and maintain the SC proliferation phase [6, 11]. The inflammatory response reaches a peak by day 3 after injury and is followed by a gradual onset of the pro-regenerative phase when pro-inflammatory cytokines (IL-1ß, TNF-α) are supplanted by pro-regenerative cytokines (IL-10, IL-6), leading to a switch of pro-inflammatory F4/80^+^Ly6C^hi^ macrophages into pro-regenerative F4/80^+^Ly6C^lo^ macrophages by day 10 after CTX injury. The switch to anti-inflammatory cytokines stimulates myoblast fusion into myofibers supporting new muscle growth. Typically, by day 30 after CTX injection the injured muscle is fully repaired [1, 11–13]. Although the events that shape the SC response to injury have been extensively studied, the enduring effects on SC behavior post-injury are not clear.

Recent studies have described a novel process, termed trained immunity or inflammatory memory, whereby exposure to inflammatory mediators leads to epigenetic reprogramming in innate immune cells [14–16]. Cytokines (IL-1β, GM-CSF and IFN-γ) and damage-associated molecular patterns (DAMPs), typically released from inflamed tissues, have all been shown to induce inflammatory adaptation in some cell types, leading to enhanced responses following subsequent insults [17–21]. This process has also been described in non-immune tissue resident cells, including epithelial stem cells [14–16, 19–23]. Whether previous muscle injury alters SC responses to subsequent injury is not known. Here, we addressed this question using a mouse model of repeated CTX injury and show that prior injury has enduring effects on SC behavior, resulting in accelerated muscle regeneration upon secondary challenge.

## Materials and methods

### Mice

C57BL/10ScSn mice were purchased from the Jackson laboratory (Bar Harbor, ME, USA). Only male mice were used. The mice were housed in the Histology Department-accredited animal facility at the University of Sapienza. The minimum number of mice per experiment necessary to obtain statistically significant data was determined using power calculations. Mice were randomly allocated to control and intervention groups. All the procedures were approved by the Italian Ministry for Health and were conducted according to the EU regulations and the Italian Law on Animal Research.

### Cardiotoxin injury

To induce acute muscle injury, tibialis anterior and gastrocnemius muscles were injected with, respectively, 0.01 ml or 0.02 ml of cardiotoxin from Naja Pallida (10 µM in H_2_O, Latoxan ZA Les Auréats, France), using a 30-gauge micro-syringe, as previously described [10, 13, 24]. For tibialis anterior, 0.01 ml of cardiotoxin was delivered in a single injection, while for gastrocnemius the 0.02 ml of cardiotoxin was administered as two 0.01 ml injections.

### Freeze injury

To perform freeze injury, mice were anesthetized with zoletil (Virbac Laboratories) (50:50 mg/ml) and rompum (Bayer) (20 mg/ml), and placed on a warm pad, and tibialis anterior was exposed by surgical incision of the skin. A sterile metal probe, pre-cooled with dry ice, was gently pressed on the muscle for 10 s. The incision was then closed using surgical glue.

### Tissue processing and cell isolation for FACS

Muscle single-cell suspensions were obtained as previously described [13, 24]. Briefly, mice were killed by cervical dislocation and perfused with PBS, and the hind limb musculature (tibialis anterior and gastrocnemius) was excised, weighed, cut into 1-mm pieces and digested with 1 mg/ml Collagenase type 4 (Worthington) for 1.5 h in a pre-warmed (37 °C) water bath with agitation. Muscles from each mouse were processed individually. The digested muscle preparation was first passed through a 70 μm cell strainer (BD Falcon) and then through a 40 μm cell strainer, washed in Dulbecco’s modified eagle’s medium (DMEM)/10% fetal bovine serum (FBS) and resuspended in FACS buffer (PBS 1%FBS). Single cells were counted with a hemocytometer and incubated with anti-CD16/32 (clone 93, Biolegend) to block non-specific binding to Fc receptors. Samples were then stained with the relevant antibodies anti-CD45 (clone 30-F11); anti-Ly6C (clone HK14); anti-Ly6g (clone 1A8); anti-F4/80 (clone BM8) all from Biolegend). Cell viability was assessed with 4′,6-diamidino-2-phenylindole, dilactate (DAPI) (Biolegend). Samples were acquired with a CyAn ADP (DAKO), and acquired data were analyzed using FlowJo software version 10.

### Histological analysis

Histological analysis was performed on frozen muscle sections as previously described [13]. Tibialis anterior muscles were embedded in tissue freezing medium (Leica) and snap-frozen in liquid nitrogen-cooled isopentane. Frozen muscles were cut into 8 μm sections and stored at − 20 °C until use. For morphological assessment, frozen sections were stained with H&E.

### Immunohistochemistry analysis

Immunofluorescence analysis was performed as described previously [10]. For immunofluorescence staining of muscle, cryosections were fixed in 4% PFA for 10 min at room temperature (RT), permeabilized in cold methanol for 6 min at − 20 °C, followed by antigen retrieval in citric acid (0.01 M, pH 6), for 10 min at 90 °C. Sections were then blocked in BSA (Sigma-Aldrich St. Louis, Missouri, USA) 4%, goat serum (Sigma-Aldrich, St. Louis, Missouri, USA) 5% for 30 min RT, followed by incubation with the relevant antibodies diluted in bovine serum albumin (BSA) 4% over night at 4 °C: mouse anti-Pax7 antibody, (1:10, Developmental Studies Hybridoma Bank, Iowa City, Iowa), rabbit anti-laminin (1:400 Sigma-Aldrich, St. Louis, Missouri, USA), rabbit anti-ki-67 (1:300, Cell Signaling, Denver, Massachusetts, USA) or rabbit anti-ps6 (1:100, Cell Signaling, Denver, Massachusetts, USA). The next day, sections were incubated with biotin anti-mouse (1:1000 Biolegend, San Diego, California) 1 h RT. The sections were then washed three times with PBS for 15 min and subsequently incubated with secondary antibodies Streptavidin-Cy3 (1:500 Biolegend) and goat anti-rabbit IgG Alexa Fluor 488 (1:1000 Abcam, Cambridge, UK).

For FAPs, macrophages and endothelial cell frozen muscle sections were fixed and incubated with CD90 (1:150, clone 30-H12, Invitrogen, USA), CD68 (1:500, clone FA-11, AbDSerotec, America) or CD31 (1:100, clone MEC 13.3, Biolegend, San Diego), respectively, followed by the relevant secondary antibodies.

For satellite cells or myotubes staining, cells grown in vitro were washed with PBS, then fixed in paraformaldehyde (PFA) 4% for 5 min RT, permeabilized in cold methanol − 20◦C for 6 min and blocked in BSA 4%, goat serum 5% for 30 min RT. Primary antibodies mouse anti-Pax7 (1:10, Developmental Studies Hybridoma Bank, Iowa City, Iowa), rabbit anti-ki-67 (1:400, Cell Signaling, Denver, Massachusetts, USA) and mouse anti-MHC were incubated O/N at 4◦C. The next day cells were incubated for 1 h at RT in biotin anti-mouse and subsequently in secondary antibodies goat anti-rabbit Alexa Fluor488 and Streptavidin-Cy3 for 1 h RT. Nuclei were counterstained with Hoechst, and samples were analyzed under an epifluorescence Zeiss Axioskop 2 Plus microscope (Carl Zeiss, Oberkochen, Germany).

The percentage of Pax7hi and Pax7lo was calculated in FACS-sorted satellite cells cytospinned onto a microscopy glass and stained for Pax7 as above. Fluorescence intensity was determined by measuring mean gray value for every Pax7^+^ cells using ImageJ built-in functions. Briefly, images with Pax7 staining were converted into 8-bit, a threshold was set, and a binary image was created and smoothed in order to clearly separate cells from background and precisely select cells perimeters. The selections were then translated to the 8-bit image using the ‘ROI manager’ function. Mean gray value was then measured for each Pax7^+^ cell. After background subtraction, an arbitrary threshold of 30 was empirically set to discriminate Pax7^hi^ and Pax7^lo^ cells.

The cross-sectional area (CSA) of regenerating fibers was examined using anti-laminin antibody (Sigma, 1:200) to visualize the fibers and Image J software for calculating the area of regeneration.

### Satellite cells isolation for cell culture

Briefly, tibialis anterior and gastrocnemius muscles were dissected with scissors and finely diced with a scalpel into 1-mm pieces. For enzymatic digestion, muscles were incubated with Collagenase type II (Sigma-Aldrich) 0.4 mg/ml in PBS (Sigma-Aldrich), for 45 min in a shaking water bath at 37^◦^C. A second digestion was performed in 1 mg/mL of Collagen/Dispase (Roche, Basel, Switzerland) in PBS calcium–magnesium-free (Sigma-Aldrich), for 30 min at 37◦C. The digested muscle was then passed through 70 μm cell strainer first and 40 μm cell strainer then to remove debris [25]. Next, satellite cell purification was performed by using SC Isolation Kit (Miltenyi Biotech, Bergisch Gladbach, Germany).

#### FACS-sorting of satellite cells

Quadriceps and gastrocnemius muscles of mice were subjected to enzymatic dissociation (in PBS with 2 mg/mL Collagenase A (Roche, Basel, Switzerland), 2.4 units/mL Dispase I (Roche, Basel, Switzerland), 10 ng/mL DNase (MilliporeSigma), 0.4 mM CaCl2 and 5 mM MgCl2) for 30 min at 37 °C. The cell suspension was filtered through a 40 μm nylon filter and incubated with the following antibodies for 30 min: CD45 (1:50, Invitrogen, USA), CD31 (1:50, Invitrogen, USA), TER119 (1:50, Invitrogen, USA), Sca1 (1:50, Invitrogen, USA), Itga7 (1:500, AbLab, USA).

Satellite cells were identified as TER119^−^CD45^−^CD31^−^Itga7^+^Sca1^−^ cells.

Cell sorting was performed with MoFlo Astrios (Beckman Coulter).

#### Satellite cell culture

Cells were then counted, washed and resuspended in growth medium (GM) containing DMEM 20% horse serum (HS) and 3% chicken embryo extract (CEE), and plated. An aliquot of the satellite cells was cyto-spined onto a microscopy glass for immunofluorescence analysis of the cells before culture. After 3 days in culture, cells were switched to differentiation medium (DM) containing DMEM 5% HS, 1% CEE.

### Real-time quantitative PCR

RNA was isolated from muscle single cells using Trizol (Sigma) according to the manufacturer's instructions. cDNA was synthesized using the High-Capacity cDNA Reverse Transcription kit (Applied Biosystems). PCR amplification was performed using the SensiMix™ SYBR® Low-ROX Kit, following the manufacturer’s protocol. Data analysis was performed 7500 Software v2.0.6 provided by Applied Biosystems. Data are expressed as fold change in expression levels. Relative expression levels were normalized to GAPDH mRNA. The following primer pairs were used for amplification: CCL-2 (forward), GTTGGCTCAGCCAGATGCA, (reverse), AGCCTACTCATTGGGATCATCTTG; IL1β (forward), AGTGTGGATCCCAAGCA, (reverse), CACTGTTGTTTCCCAGGA; IL10 (forward), CAAAGGACCAGCTGGACA, (reverse), ATCGATGACAGCGCCTCA; IL-6 (forward), ATG AAGTTCCTCTCTGCAAGAGACT, (reverse), CACTAGGTTTGCCGAGTAGATCTC; Pax7 (forward), GTCCCAGTCTTACTGCCAC, (reverse), TGTGGACAGGCTCACGTTTT. M-cadherin (forward), CCTACCCACTTGTGCAGATCA, (reverse), TGGAGAAGACATTTCGGGGC; IL6-receptor (forward), TCACTGTGCGTTGCAAACAG, (reverse), GATCCGGCTGCACCATTTTT; CXCR4 (forward), TCAGTGGCTGCCTCTT, (reverse), CTTGGCCTTTGTTGGT; VCAM-1 (forward), GCACTCTACTGCGCATCTT, (reverse), CACCAGACTGTACGATCCT.

### Single-cell reanalysis

Original data from Oprescu et al. [26] were downloaded from GEO repository (accession number GSE138826). RNA counts and metadata (including original cell annotation) were imported from the regendata.rds file containing the merged dataset with all cells from all time points. Data were processed and normalized as previously described in the original paper with minor modifications (SCTransform v2 function) [27]. The top 30 principal components were used for UMAP embedding.

### Statistical analysis

All statistical analyses were performed using GraphPad Prism software version 6. Data are presented as mean ± SEM. Statistical significance was determined using unpaired two-tailed Student's *t* test with Welch's correction for unequal variances. A* P* value of < 0.05 was considered statistically significant.

## Results

### Injury-experienced satellite cells show accelerated cell cycle entry

**Fig. 1:**
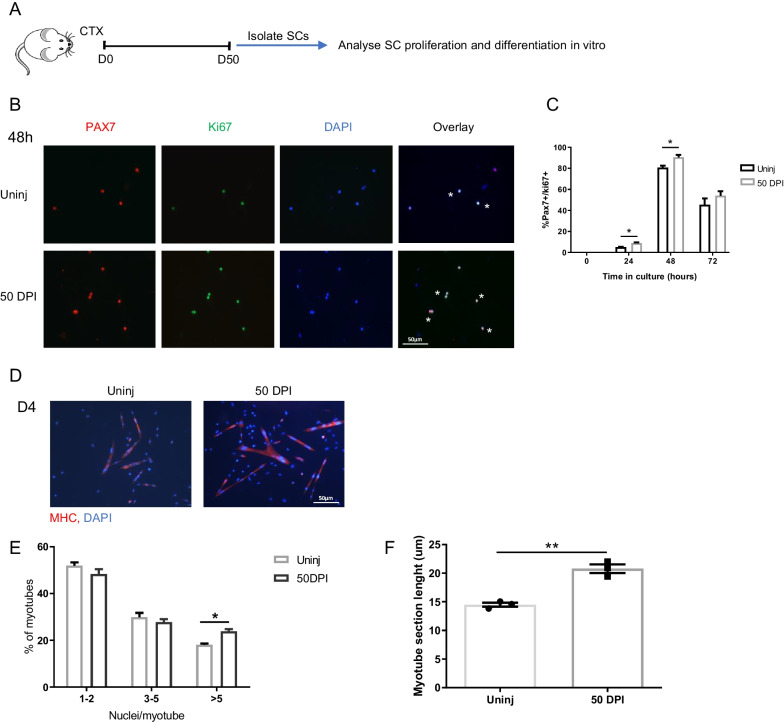
Injury-experienced SCs proliferate sooner and form bigger myotubes in vitro: **A** schematic diagram of the experimental approach. **B** Representative images of Pax7^+^/ki-67^+^ SCs, isolated from uninjured muscle or 50 days after cardiotoxin injury (50 DPI) and cultured for 48 h. **C** Quantification of Pax7^+^/ki-67^+^ SCs as in **B**. **D** Representative images of MHC^+^ myotubes formed by SCs isolated 50 DPI or control and cultured in growth medium for 72 h followed by additional 24 h in differentiation medium. **E** Quantification of the percentage of myotubes with 2 or less, 3 to 5, or more than 5 nuclei. **H** Quantification of myotube size, expressed as medium diameter in microns. *n* = 3 independent samples. Data are shown as mean ± S.E.M. *p < 0.05; **p < 0.01

To explore the possibility that inflammatory adaptation, a process which has been described in various cell types [14, 19, 28, 29], might also be a feature of skeletal muscle satellite cells (SCs), we injured the gastrocnemius (GA) and tibialis anterior (TA) muscles of WT mice with CTX injection, and 50 days after the CTX injury (50 DPI), when the muscle is fully recovered (Additional file 1: Fig. S1A–C), we isolated the SCs (from now on referred to as injury-experienced SCs, ieSCs) to examine their rate of proliferation and ability to form myotubes ex vivo (Fig. 1A). Neither ieSCs, nor control SCs isolated from uninjured muscle (from the uninjured contralateral leg), were positive for the proliferation marker ki67 at the time of isolation (T0), and there was no difference in their number or size (Additional file 2: Fig. S2A–C). On the other hand, when cultured in vitro, a significantly higher percentage of ieSCs were positive for Ki67 at 24 and 48 h after plating, compared to control SCs, suggesting that ieSCs enter the cell cycle faster than injury inexperienced control SCs (Fig. 1B–C). Moreover, after 4 days in culture, ieSCs formed bigger myotubes with increased number of nuclei compared to control SCs, suggesting that they fuse at a significantly higher rate (Fig. 1D–F). These results show that previous injury has a lasting effect on SC behavior that is independent from the muscle microenvironment.

### Activation of the mTORC 1 signaling pathway is a feature of injury-experienced SCs

**Fig. 2:**
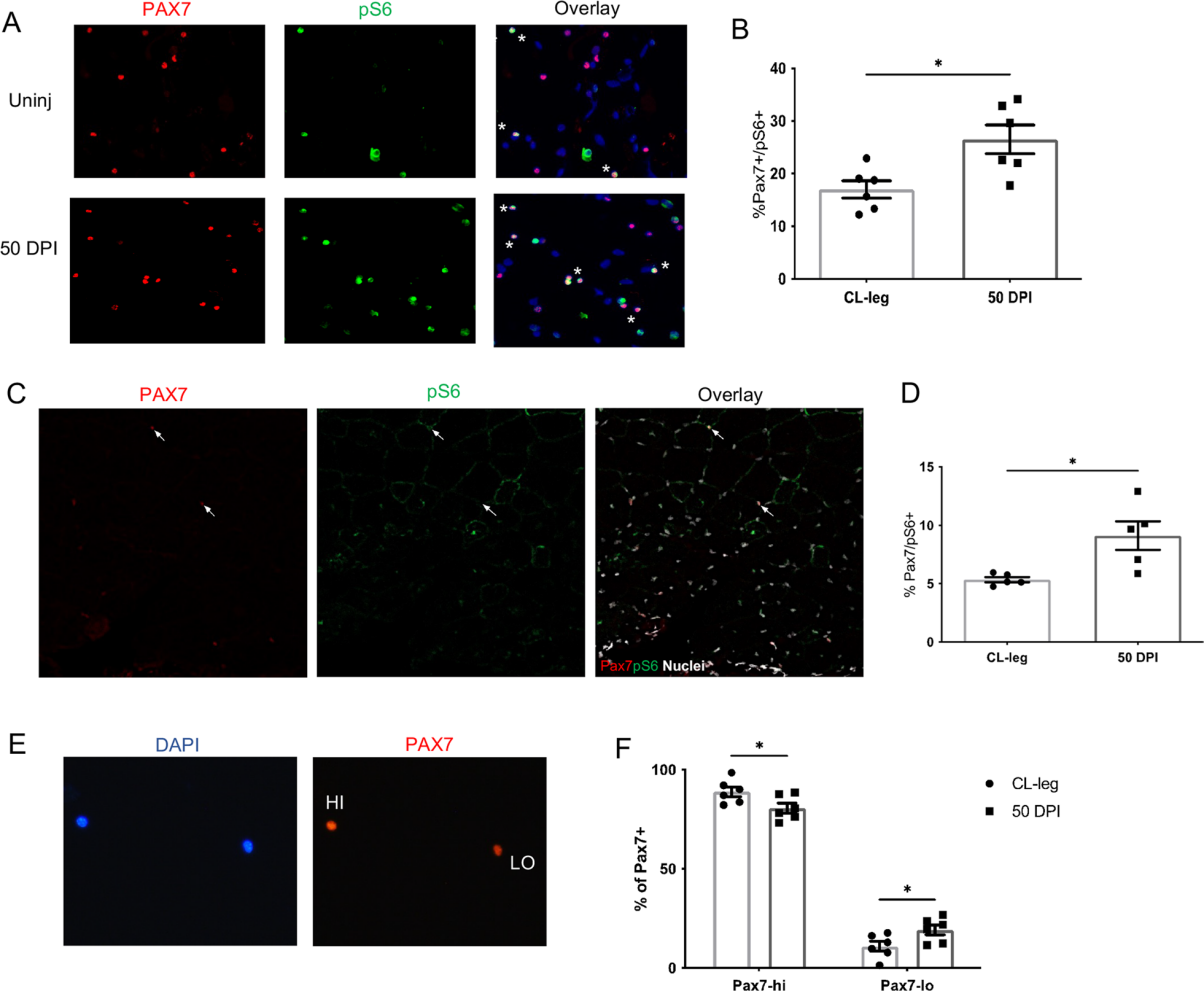
Satellite cells retain S6 phosphorylation 50 days after cardiotoxin injury. **A** Representative images of Pax7^+^/pS6^+^ SCs isolated from uninjured muscle or 50 DPI. **B** Quantification of the percentage of pS6^+^ SCs. *n* = 6 independent samples. **C** Representative Pax7^+^/pS6^+^ and Pax7^+^/pS6^−^ SCs in a TA muscle section. Arrows indicate a Pax7^+^/pS6^+^ SC (upper arrow) and a Pax7^+^/pS6^−^ SC (lower arrow). **D** Quantification of the percentage of pS6^+^ SCs in muscle sections 50 DPI, or in uninjured control muscle. *n* = 6 independent samples. **E** Representative images of Pax7^hi^ and Pax7^lo^ cells isolated from muscle 50 DPI or from uninjured control muscle. **F** Quantification of the percentage of Pax7^hi^ and Pax7^lo^ SCs isolated from muscle 50 DPI or from uninjured control muscle. *n* = 5 independent samples. Data are shown as mean ± S.E.M. *p < 0.05

A previous work by Rodgers JT et al. [30] observed that SCs located distal to the site of injury (uninjured contralateral leg) can transition from a quiescent to an alert state, enabling them to respond rapidly following injury, and that this event was dependent on mTORC1 signaling. To examine whether a similar mechanism governs the enhanced SC responses in our model, we isolated SCs from muscle 50 DPI and analyzed the expression of phospho-S6 (pS6), which is the downstream target of mTOR signaling. As shown in Fig. 2A, B, a significantly higher number of ieSCs were pS6+ compared to control SCs isolated from uninjured muscle. Accordingly, immunofluorescence on muscle sections at 50 DPI revealed a significantly increased fraction of SCs expressing pS6 compared to SC in control uninjured muscle (Fig. 2C, D).

Quiescent muscle stem cells can exist in states that range from deep quiescence to a poised state and can be discriminated by the level of Pax7 expression [31]. Pax7^hi^ cells exist in deep state of quiescence or dormant state, whereas Pax7^lo^ cells are more poised to activate faster. Thus, Pax7^hi^ cells take longer to enter the cell cycle and have lower mitochondrial activity compared to Pax7^lo^ cells [31–33]. Interestingly, we found increased frequency of Pax7^lo^ cells among ieSCs isolated at 50 DPI compared to uninjured muscle (Fig. 2E, F), suggesting that ieSCs are poised to activate faster.

### Previous experience of injury enables satellite cells to regenerate muscle faster

**Fig. 3:**
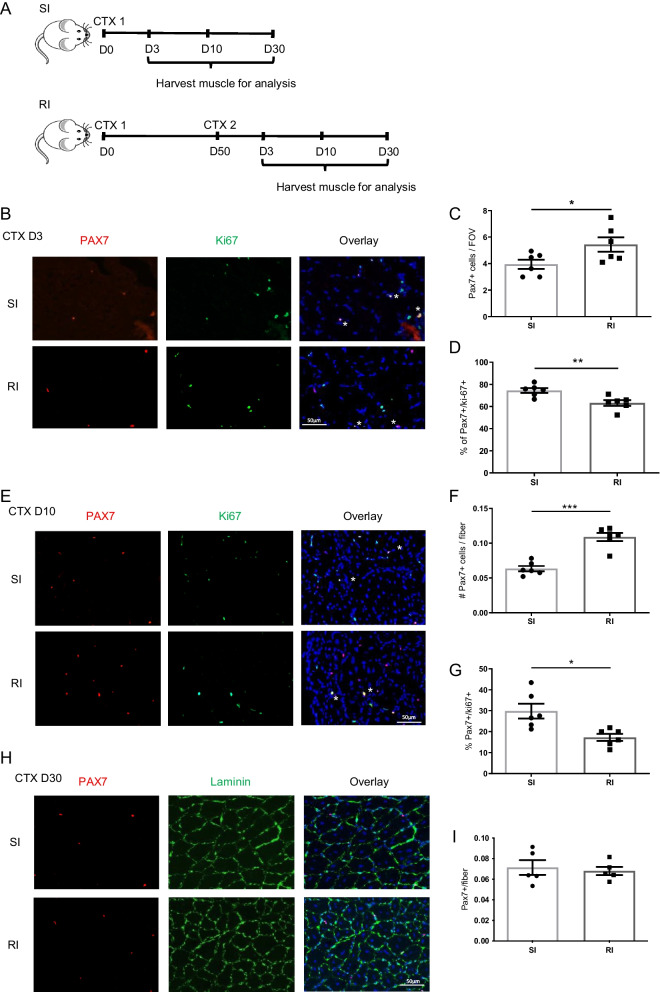
Pax7^+^ cells are increased 3 and 10 days after repeated injury: **A** Schematic diagram of the experimental approach. **B** Representative images of proliferating Pax7^+^/ki-67^+^ SCs in muscle sections, 3 days after single or repeated injury. **C** and **D** Quantification of the number of Pax7^+^ cells per field of view (FOV) and of the percentage of Pax7^+^/ki-67^+^ SCs in muscle sections. *n* = 6 independent samples. **E** Representative images of Pax7^+^/ki-67^+^ SCs in muscle sections, 10 days after single or repeated injury. **F** and **G** Quantification of the percentage of Pax7^+^/ki-67^+^ SCs and the number of Pax7^+^ cells per muscle fiber. *n* = 6 independent samples. **H** Representative images of Pax7^+^ cells in muscle sections, 30 days after single or repeated injury. **I** Quantification of the number of Pax7^+^ cells per muscle fiber. *n* = 5 independent samples. Data are shown as mean ± S.E.M. *p < 0.05; **p < 0.01; ***p < 0.001

Having demonstrated that ieSCs are poised to activate and enter the cell cycle faster compared to injury-naïve SCs, we wondered if these properties of ieSCs are associated with altered kinetics of skeletal muscle regeneration following secondary injury. To this aim, we injected the gastrocnemius and tibialis anterior muscles of WT mice with CTX, and 50 days later, at a time when the muscle is fully recovered (Additional File 1: Fig. S1A), we administered a second CTX injection (Fig. 3A). Muscle was harvested for analyses at 3-, 10- and 30- DPI, with 3 DPI representing the peak in inflammation and SC proliferation, 10 DPI the peak of regeneration and 30 DPI the completion of the repair process and return to homeostasis. We found that the number of Pax7^+^ SCs in muscle at 3 (Fig. 3B, C) and 10 DPI (Fig. 3E, F) after the second injury was significantly increased compared to single injury. In line with these findings, RT-qPCR performed on isolated muscle mononuclear cells revealed an increase in Pax7 mRNA in muscle at 3 and 10 DPI after repeated injury compared to single injury (Fig. S3A). Similarly, significantly more SCs were FACS-sorted from muscle at 3 DPI after the secondary injury (Additional file 3: Fig. S3B and C). Interestingly, and in line with our in vitro data on ieSCs proliferation, the percentage of SCs positive for the proliferation marker Ki67 at 3 and 10 DPI after secondary injury was significantly lower compared to single injury (Fig. 3D and G), suggesting that SCs complete their proliferation cycle earlier following the second injury. By day 30, the number of SCs was similar in muscles following single and repeated injury (Fig. 3H, I).

Next, we wondered if the expression level of chemokines and cytokine receptors as well as adhesion molecules (including CXCR4, VCAM-1, M-cadherin and Il6-r) is altered in SCs upon secondary injury. The chemokine receptor CXCR4 is known to regulate SC activation, migration and early expansion following muscle injury [34], while M-cadherin expression controls SC adhesion and cell-to-cell interactions as well as myoblast fusion [35]. Interestingly, RT-qPCR analysis showed significantly higher expression levels of M-cadherin and IL6-r in SCs isolated from muscle at 3 days after the second injury compared to single injury (Additional file 3: Fig. S3D).

Collectively, these data support our in vitro data showing that ieSCs activate and proliferate sooner.

**Fig. 4:**
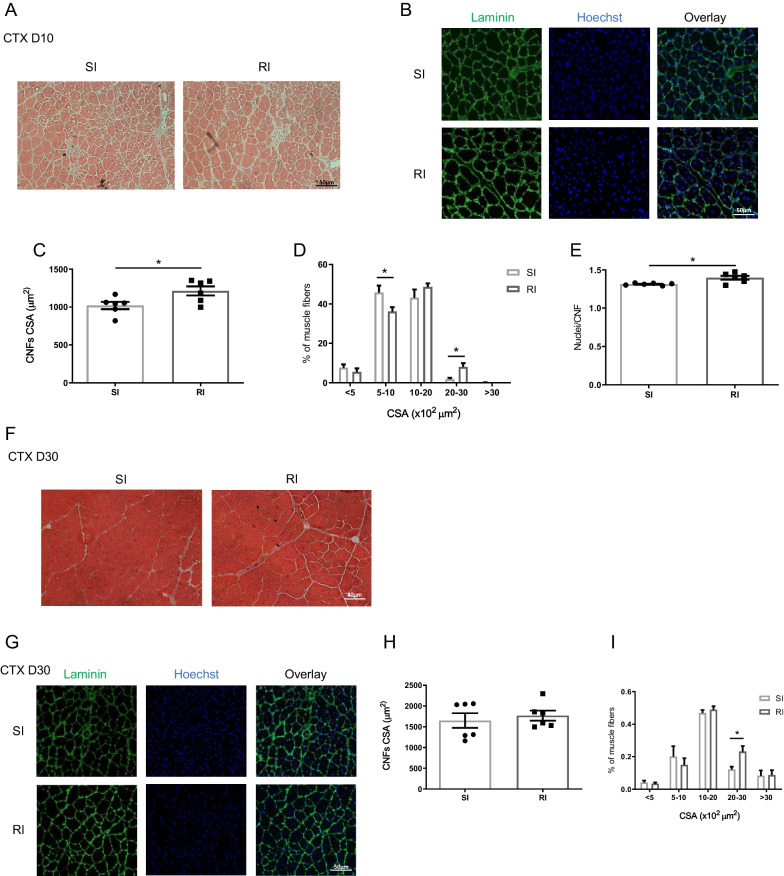
Accelerated muscle regeneration after repeated cardiotoxin injury: **A** and **B** Representative images of centrally nucleated regenerating fibers (CNFs), evidenced by H&E (**A**) or laminin staining (**B**), in muscle sections, 10 days after single or repeated cardiotoxin injury. **C** and **D** Quantification of the mean cross-sectional area (CSA) of the CNFs, expressed in square microns, and quantification of the distribution of CNFs per CSA, expressed in percentage. **E** Quantification of the number of nuclei per CNF in muscle sections. **F** and **G** Representative images of CNFs, evidenced by H&E (**F**) or laminin staining (**G**), in muscle sections, 30 days after single or repeated cardiotoxin injury. **H** and **I** Quantification of mean CSA of the CNFs, expressed in square microns, and quantification of the distribution of CNF per CSA, expressed in percentage. *n* = 6 independent samples. Data are shown as mean ± S.E.M. *p < 0.05

The kinetics of activated, proliferating and differentiating SCs, is closely associated with the rate and quality of myofiber regeneration [1]. We therefore examined whether the increased number of SCs after repeated injury results in enhanced regeneration. At 10 DPI, most of the fibers within the injured area were centrally nucleated (CNF) signifying ongoing regeneration, and there was no difference in the number of regenerating myofibers between single and repeated injury (data not shown). However, analysis of the cross-sectional area (CSA) of regenerating fibers revealed a significantly increased mean CSA after repeated injury compared to single injury (Fig. 4A–C). Furthermore, the presence of fibers with CSA greater than 3000 μm at 10 DPI was significantly higher following repeated injury, whereas fibers smaller than 500 μm were significantly less compared to single injury (Fig. 4D). Likewise, repeated injury was associated with increased number of nuclei per fiber compared to single injury (Fig. 4E). Taken together, these observations suggest that muscle regenerates faster following a second injury, probably due to enhanced myoblast fusion. By day 30, when regeneration is complete, these differences were no longer apparent (Fig. 4F–I). Interestingly, fibers with CSA of 3000 µm remained significantly higher in muscle at 30 DPI following repeated compared to single injury or uninjured control muscle, suggesting a regenerative advantage even at later time points. This enhanced regenerative capacity of ieSCs was independent from the type of injury since we obtained similar results upon repeated freeze injury (Additional File 4: Fig S4A–H).

### Previous injury conditions the muscle microenvironment and boosts cytokine levels upon subsequent injury

Satellite cells’ ability to regenerate injured muscle is strongly dependent on cues from the muscle microenvironment such as pro- and anti-inflammatory cytokines released from muscle resident cells and infiltrating immune cells. We therefore performed flow-cytometric analysis to examine the number and type of infiltrating immune cells, and immunofluorescence analysis on muscle sections to quantify the resident immune cells such as CD68+ macrophages, fibro-adipogenic progenitors (FAPs) and endothelial cells following secondary injury. We found no difference in the number of CD45^+^ cells recruited to injured muscle (Additional file 5: Fig. S5A), including Ly6g+ neutrophils, F4/80^+^Ly6C^hi^ inflammatory monocytes/macrophages (Additional file 5: Fig. S5B and C). Similarly, there was no difference in the number and percent of proliferating FAPs, or in the number of CD68+ macrophages at 7 DPI (Additional file 6: Fig. S6A–E) following single and repeated injury as assessed by immunofluorescence.

An increase in angiogenesis could influence the kinetics of muscle regeneration after injury. We therefore examined the number of CD31+ cells and percent of proliferating CD31+ cells in muscle sections at 7 and 30 days after single and repeated injury. We found no difference in the number or proliferation of CD31+ cells at 7DPI and, as expected, no difference in their number at 30 DPI compared to uninjured muscle (Additional file 7: Fig. S7A–D).

**Fig. 5:**
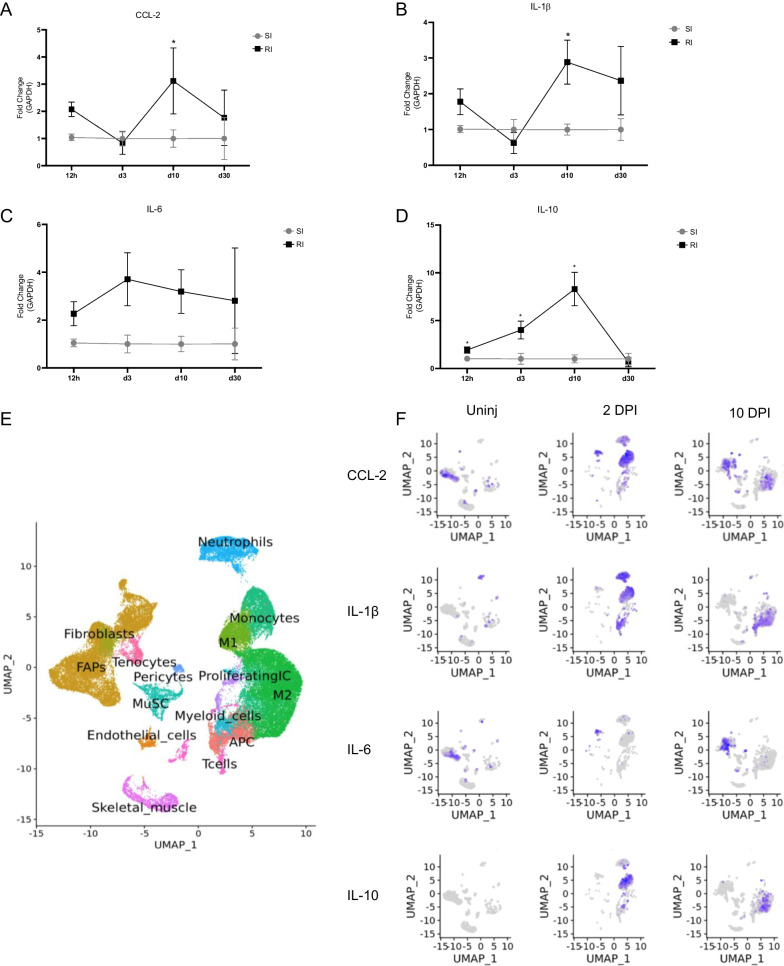
Differential cytokines expression in muscle upon repeated cardiotoxin injury: **A**–**D** Quantification of CCL-2 (**A**), IL-1β (**B**), IL-6 (**C**) and IL10 (**D**) relative expression as measured by RT-qPCR. RNA was extracted from mononuclear cells isolated from muscle at 18 h, 3 days, 10 days and 30 days after single or repeated cardiotoxin injury. Data are expressed as fold change (2-ddCT) normalized against GAPDH. Data are shown as mean ± S.E.M. *p < 0.05. **E** UMAP plot of whole-muscle single-cell RNAseq database from Oprescu et al. [26]. **F** Single-cell expression patterns of CCL2, IL-1β, IL-6 and IL10 in injured (2 or 10 days post-cardiotoxin) or uninjured control (from Oprescu et al. [26])

By contrast, RT-qPCR analysis revealed that although the kinetics of cytokine expression after single and repeated injury is similar (data not shown), we observed a significantly greater level of chemokine and cytokine expression at 12 h, day 3 and day 10 after the repeated compared to single injury, although these differences were no longer observed at day 30 after injury (Fig. 5A–D). In particular, there was an increase in the level of the monocyte recruitment chemokine CCL-2, the pro-inflammatory cytokine IL1-β and the pro-regenerative cytokine IL-10 after repeated compared to single injury (Fig. 5A–D). The level of IL-6 was also increased following repeated injury, although this increase did not reach statistical significance. Therefore, although the number of resident and recruited inflammatory cells remained unchanged after secondary damage, it is possible that previous injury boosted cytokine release from those cells. To determine the cell source of these cytokines in injured muscle, we reanalyzed the publicly available transcriptome data from Oprescu et al. [26] of spatiotemporal dynamics in gene expression, population composition and cell-to-cell interaction during muscle regeneration at 2 and 10 DPI (Fig. 5E, F).

As expected, monocyte-derived macrophages are the main source of CCL-2, IL-1β and Il-10 at 2 and 10 DPI. Interestingly, at the height of regeneration, during the acute phase of injury at 10 DPI, FAPs are the major source of the monocyte chemotactic protein CCL-2 and of IL-6. Although we found no change in the number of FAPs or macrophages, these data suggest that these cell populations might also be subject to inflammatory adaptation whereby prior inflammatory response during muscle injury primes them to produce higher level of cytokines upon subsequent injury.

## Discussion

Niche adaptation or inflammatory memory can arise in response to a broad set of environmental stimuli and lead to a general state of hyper-responsiveness, which can then alter the response upon secondary exposure [22, 36]. This process is mediated by epigenetic changes in chromatin accessibility such that a particular gene expression pattern is induced upon secondary stimulation, which allows a faster and stronger response to subsequent challenge compared to the primary inflammatory event [15, 19].

Here, we demonstrate for the first time that the effect of previous injury on the muscle stem cells, the SCs, is long-lived, leading to quantitative and qualitative changes in SC responses to secondary injury, and resulting in enhanced regeneration.

The injury-experienced SCs were poised to activate sooner after second injury, fused earlier to form big myofibers during regeneration and completed the process ahead of injury naïve SCs.

Among the defining features of ieSCs were increased activation of the mTORC1 signaling pathway and reduced expression of Pax7, which likely allow them to respond faster following injury. Similar to our findings in the injured muscle, Rodgers JT et al. [30] demonstrated that SCs located distal to the site of injury (uninjured contralateral leg) can transition from a quiescent to an alert state, enabling them to respond rapidly following injury. However, there are obvious and important differences between our and Rodgers JT et al. approach. While we investigated the enduring consequences of acute injury within the injured muscle and waited for the muscle to fully recover (50 days) before examining the changes in response to secondary injury within the same muscle, Rodgers JT et al. [30] examined the SCs in the contralateral uninjured leg and compared them to the SCs in the injured leg during the early response to a single injury. Following muscle injury (at 3 DPI), SCs from the contralateral uninjured limb muscle showed a higher propensity to enter the cell cycle when compared to quiescent SCs without proceeding to an activation stage, which was dependent on mTORC1 signaling. By 28 days post-injury the alert SCs from the contralateral leg returned to quiescence. Indeed, in our model, at 50 days after a single injury in one leg, regeneration in the contralateral leg was not enhanced in response to injury suggesting that the alert state of SCs in the contralateral leg is short-lived, most likely transiently activated because of the increase in inflammatory cytokines released in circulation following injury.

Given the fact that in our model the first injury occurred 50 days earlier, and the muscle was fully recovered by the time of the second injury, our results suggest that the muscle can ‘learn’ from a previous injury, to hasten recovery upon secondary challenge. In addition to what we described in the SC, the priming process during injury might occur across various cell components within the muscle, or indeed recruited immune cells, remembering different aspects of the initial injury, and cooperating during the secondary injury to ensure faster and more efficient regeneration. In support of this hypothesis, we found a significant increase in pro- and anti-inflammatory cytokines, such as IL-6, IL-1b and IL-10 in muscle following secondary injury, likely produced by FAPs and monocyte-derived M1 and M2 macrophages. These cytokines are known to contribute to both the early pro-inflammatory response to injury, by stimulating SC proliferation, but also to the regeneration phase, by stimulating myoblast fusion and the formation of new fibers [1, 11, 13]. It is therefore conceivable that the increased levels of cytokines and chemokines produced by immune and non-immune cell components of the muscle SC niche would further enhance the regenerative potential of ieSCs. Interestingly, a recent work by Bhattarai et al. [37] showed that bone marrow-derived macrophages isolated from a murine model of Duchenne muscular dystrophy (DMD), which is characterized by chronic muscle injury exhibit cardinal features of trained immunity, including increased cytokine production, changes in cell metabolism and epigenetic remodeling.

Although largely descriptive at present, our study highlights the ability of muscle to respond faster and more efficiently following secondary injury, raising a number of fascinating questions to explore in future research.

Ongoing work in our laboratory will seek to identify the cell population(s) other than SCs that undergo priming following injury, the transcriptional and epigenetic changes enabling them to hasten recovery following secondary injury, and how they may cooperate. Another obvious question arising from our study pertains to the physiological relevance of this phenomenon in muscle. Is it beneficial to the organism as a whole, and might this seemingly beneficial process be detrimental in the settings of chronic injury and inflammation in muscle diseases?

## Conclusion

In this study, we show that skeletal muscle injury has a long-lasting effect on the muscle stem cells, the SCs, such that ieSCs demonstrate enhanced regenerative properties upon subsequent injury resulting in accelerated regeneration. Future studies are needed to understand the long-term health ramifications of this process and uncover novel potential avenues to explore in order to harness its beneficial effects to ameliorate muscle regeneration in muscle diseases.

### Supplementary Information

**Additional file 1: Fig. S1**: A) Representative images of uninjured muscle (uninj) and 50 days post-cardiotoxin injury (50 DPI) stained by hematoxylin and eosin (H&E), showing complete fiber regeneration by 50 days after injury. B) Representative images of Pax7 and laminin staining in uninjured muscle or 50 days after cardiotoxin injection. C) Quantification of the number of Pax7^+^ cells per muscle fiber as in (B). n = 3 independent samples. Data are shown as mean ± S.E.M.

**Additional file 2: Fig. S2**: A) Quantification of the number of Pax7^+^ cells per field of view (FOV) after SCs isolation from injured (50 days post-injury (50DPI)) or uninjured muscle assessed by immunofluorescence. B) Quantification of the percentage of Pax7^+^/ki-67^+^ SCs isolated 50 DPI or uninjured muscle assessed as in (A). C) Quantification of cell diameter, expressed in microns, of the SCs isolated 50 DPI or uninjured muscle. n = 3 independent samples. Data are shown as mean ± S.E.M.

**Additional file 3: Fig. S3**: A) Quantification of Pax7 relative expression, as measured by RT-qPCR. RNA was extracted from mononuclear cells isolated from muscle 18 h, 3 and 10 days after single or repeated cardiotoxin injury. Data expressed as fold change (2-ddCT) normalized against GAPDH. B) Representative images of the gating strategy for the sorting of SCs, FAPs and macrophages from muscle. C) Total number of SCs sorted from muscle following single or repeated injury. Quantification of VCAM-1, CXCR4, M-cadherin and IL6-r relative expression by RT-qPCR in SCs FACS-sorted from muscle at 3 DPI following single or repeated injury. Data are shown as mean ± S.E.M. * = p < 0.05, * = p < 0.01.

**Additional file 4: Fig. S4** A) Schematic diagram of the experimental approach for freeze injury (FI). B) Representative images of Pax7/ki-67 staining of tibialis anterioris muscle sections 10 days after single (FI 1) or repeated freeze injury (FI 2). C-D) Quantification of the number of Pax7^+^ cells per muscle fiber and quantification of the percentage of Pax7^+^/ki-67^+^ SCs in muscle sections, as in (B). E) Representative images of centrally nucleated fibers (CNFs), evidenced by laminin staining, in muscle sections, 10 days after single or repeated freeze injury. F-G) Quantification of the cross-sectional area of the CNFs, expressed in square microns, and quantification of the number of nuclei per CNF, as in (D). F) Quantification of the distribution of CNFs per CSA, expressed in percentage, as in (G). 10 days after single or repeated freeze injury, expressed in percentage. N = 5 independent samples. Data are shown as mean ± S.E.M. * p < 0.05, ** p < 0.01.

**Additional file 5: Fig. S5**: A) Representative images of FACS gating strategy for the analysis of immune cells in muscle after single or repeated injury. B) Quantification of the number of hematopoietic (CD45^+^) cells infiltrating the muscle, 18 h, 3 days and 10 days after cardiotoxin injury, or uninjured muscle, and normalized per gram of tissue. C) Quantification of the number of recently recruited inflammatory monocytes/macrophages (F4/80 + Ly6C^hi^) and neutrophils (Ly6g^+^) infiltrating the muscle 3 days after cardiotoxin injury, or uninjured control muscle, normalized per gram of tissue. Data are shown as mean ± S.E.M.

**Additional file 6: Fig. S6**: A) Representative images of CD90 staining of FAPs in tibialis anterior 7 days after single or repeated injury. B) Quantification of CD90+ cells per FOV, n = 5 independent samples. C) Representative images of CD68+ macrophages staining of tibialis anterior 10 days after single or repeated injury. D) Quantification of the number of macrophages per field of view (FOV). n = 3 (SI) and 4 (RI) independent samples.

**Additional file 7. Fig. S7**: A) Representative images of CD31/ki-67 staining of endothelial cells in tibialis anterior muscle sections 7 days after single or repeated injury. B) Quantification of the number of CD31+ cells per fiber and the percentage of CD31+/ki67+ cells in muscle sections, as in (B). C) Representative images of CD31 and laminin staining of tibialis anterior muscle sections 30 days after single or repeated injury. D) Quantification of the number of CD31+ cells per fiber, as in (B).

## Data Availability

All data generated and analyzed during this study are available from the corresponding author upon reasonable request.

## Abbreviations

BSA: Bovine serum albumin
CTX: Cardiotoxin
CEE: Chicken embryo extract
CSA: Cross-sectional area
CNF: Centrally nucleated fibers
DAMPs: Damage-associated molecular patterns
DM: Differentiation medium
DMEM: Dulbecco’s modified eagle’s medium
DPI: Days post-injury
DMD: Duchenne muscular dystrophy
FAPs: Fibro-adipogenic progenitors
FBS: Fetal bovine serum
GM-CSF: Granulocyte monocyte colony stimulating factor
GM: Growth medium
GA: Gastrocnemius
HS: Horse serum
ieSCs: Injury-experienced SCs
IL: Interleukin
IFN: Interferon
PFA: Paraformaldehyde
RT-qPCR: Real-time quantitative PCR
SCs: Satellite cells
TNF-α: Tumor necrosis factor α
TA: Tibialis anterior

## References

[1] Tidball JG (2011). Mechanisms of muscle injury, repair, and regeneration. Compr Physiol.

[2] Renzini A, Riera CS, Minic I, D’ercole C, Lozanoska-ochser B, Cedola A (2021). Metabolic remodeling in skeletal muscle atrophy as a therapeutic target. Metabolites.

[3] Wang YX, Rudnicki MA (2012). Satellite cells, the engines of muscle repair. Nat Rev Mol Cell Biol.

[4] Relaix F, Zammit PS (2012). Satellite cells are essential for skeletal muscle regeneration: The cell on the edge returns centre stage. Development.

[5] Chang NC, Rudnicki MA (2014). Satellite cells: the architects of skeletal muscle. Curr Top Dev Biol.

[6] Tidball JG (2017). Regulation of muscle growth and regeneration by the immune system. Nat Rev Immunol.

[7] Farup J, Madaro L, Puri PL, Mikkelsen UR (2015). Interactions between muscle stem cells, mesenchymal-derived cells and immune cells in muscle homeostasis, regeneration and disease. Cell Death Dis.

[8] Murphy MM, Lawson JA, Mathew SJ, Hutcheson DA, Kardon G (2011). Satellite cells, connective tissue fibroblasts and their interactions are crucial for muscle regeneration. Development.

[9] Fiore PF, Benedetti A, Sandonà M, Madaro L, De Bardi M, Saccone V (2020). Lack of PKCθ promotes regenerative ability of muscle stem cells in chronic muscle injury. Int J Mol Sci.

[10] Benedetti A, Fiore PF, Madaro L, Lozanoska-Ochser B, Bouché M (2020). Targeting pkcθ promotes satellite cell self-renewal. Int J Mol Sci.

[11] Tidball JG (2005). Inflammatory processes in muscle injury and repair. Am J Physiol Regul Integr Comp Physiol.

[12] Arnold L, Henry A, Poron F, Baba-Amer Y, Van Rooijen N, Plonquet A (2007). Inflammatory monocytes recruited after skeletal muscle injury switch into antiinflammatory macrophages to support myogenesis. J Exp Med.

[13] Rizzo G, Di Maggio R, Benedetti A, Morroni J, Bouche M, Lozanoska-Ochser B (2020). Splenic Ly6Chi monocytes are critical players in dystrophic muscle injury and repair. JCI Insight.

[14] Netea MG, Joosten LAB, Latz E, Mills KHG, Natoli G, Stunnenberg HG (2016). Trained immunity: a program of innate immune memory in health and disease. Science.

[15] Van Der Heijden CDCC, Noz MP, Joosten LAB, Netea MG, Riksen NP, Keating ST. Epigenetics and trained immunity. Antioxidants Redox Signal. 2018;29(11):1023–40. 10.1089/ars.2017.7310 PMC6121175 28978221

[16] Dominguez-Andres J, Netea MG (2019). Long-term reprogramming of the innate immune system. J Leukoc Biol.

[17] Mitroulis I, Ruppova K, Wang B, Chen LS, Grzybek M, Grinenko T (2018). Modulation of myelopoiesis progenitors is an integral component of trained immunity. Cell.

[18] Borriello F, Galdiero MR, Varricchi G, Loffredo S, Spadaro G, Marone G (2019). Innate immune modulation by GM-CSF and IL-3 in health and disease. Int J Mol Sci.

[19] Naik S, Larsen SB, Gomez NC, Alaverdyan K, Sendoel A, Yuan S (2017). Inflammatory memory sensitizes skin epithelial stem cells to tissue damage. Nature.

[20] Ordovas-Montanes J, Dwyer DF, Nyquist SK, Buchheit KM, Vukovic M, Deb C (2018). Allergic inflammatory memory in human respiratory epithelial progenitor cells. Nature.

[21] Wendeln AC, Degenhardt K, Kaurani L, Gertig M, Ulas T, Jain G (2018). Innate immune memory in the brain shapes neurological disease hallmarks. Nature.

[22] de Laval B, Maurizio J, Kandalla PK, Brisou G, Simonnet L, Huber C (2020). C/EBPβ-Dependent Epigenetic Memory Induces Trained Immunity in Hematopoietic Stem Cells. Cell Stem Cell.

[23] Liu GY, Liu Y, Lu Y, Qin YR, Di GH, Lei YH (2016). Short-term memory of danger signals or environmental stimuli in mesenchymal stem cells: Implications for therapeutic potential. Cell Mol Immunol.

[24] Lozanoska-Ochser B, Benedetti A, Rizzo G, Marrocco V, Di Maggio R, Fiore P (2018). Targeting early PKCθ-dependent T-cell infiltration of dystrophic muscle reduces disease severity in a mouse model of muscular dystrophy. J Pathol.

[25] Benedetti A, Cera G, De Meo D, Villani C, Bouche M, Lozanoska-Ochser B (2021). A novel approach for the isolation and long-term expansion of pure satellite cells based on ice-cold treatment. Skelet Muscle.

[26] Oprescu SN, Yue F, Qiu J, Brito LF, Kuang S (2020). Temporal dynamics and heterogeneity of cell populations during skeletal muscle regeneration. iScience..

[27] Hao Y, Hao S, Andersen-Nissen E, Mauck WM, Zheng S, Butler A (2021). Integrated analysis of multimodal single-cell data. Cell.

[28] Larsen SB, Cowley CJ, Sajjath SM, Barrows D, Yang Y, Carroll TS (2021). Establishment, maintenance, and recall of inflammatory memory. Cell Stem Cell.

[29] Gonzales KAU, Polak L, Matos I, Tierney MT, Gola A, Wong E (2021). Stem cells expand potency and alter tissue fitness by accumulating diverse epigenetic memories. Science.

[30] Rodgers JT, King KY, Brett JO, Cromie MJ, Charville GW, Maguire KK (2014). MTORC1 controls the adaptive transition of quiescent stem cells from G 0 to GAlert. Nature.

[31] Rocheteau P, Gayraud-Morel B, Siegl-Cachedenier I, Blasco MA, Tajbakhsh S (2012). A subpopulation of adult skeletal muscle stem cells retains all template DNA strands after cell division. Cell.

[32] Olguin HC, Olwin BB (2004). Pax-7 up-regulation inhibits myogenesis and cell cycle progression in satellite cells: a potential mechanism for self-renewal. Dev Biol.

[33] Wu R, Li H, Zhai L, Zou X, Meng J, Zhong R (2015). MicroRNA-431 accelerates muscle regeneration and ameliorates muscular dystrophy by targeting Pax7 in mice. Nat Commun.

[34] Shams AS, Arpke RW, Gearhart MD, Weiblen J, Mai B, Oyler D (2022). The chemokine receptor CXCR4 regulates satellite cell activation, early expansion, and self-renewal, in response to skeletal muscle injury. Front Cell Dev Biol.

[35] Taylor L, Wankell M, Saxena P, McFarlane C, Hebbard L (2022). Cell adhesion an important determinant of myogenesis and satellite cell activity. Biochim Biophys Acta Mol Cell Res.

[36] Natoli G, Ostuni R (2019). Adaptation and memory in immune responses. Nat Immunol.

[37] Bhattarai S, Li Q, Ding J, Liang F, Gusev E, Lapohos O (2022). TLR4 is a regulator of trained immunity in a murine model of Duchenne muscular dystrophy. Nat Commun.

