# Supplementary figures and images for: Injury-experienced satellite cells retain long-term enhanced regenerative capacity
Source: Stem Cell Res Ther. 2023 Sep 12;14:246. doi: 10.1186/s13287-023-03492-4 (PMC10496398; doi:10.1186/s13287-023-03492-4)

Supplementary Figure 1

A

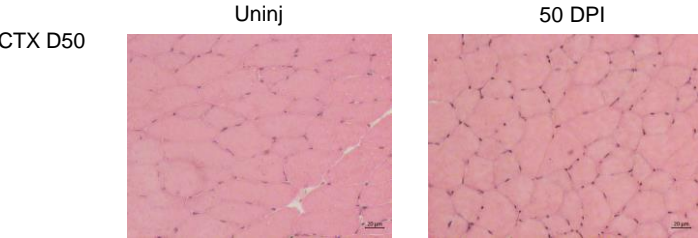

B

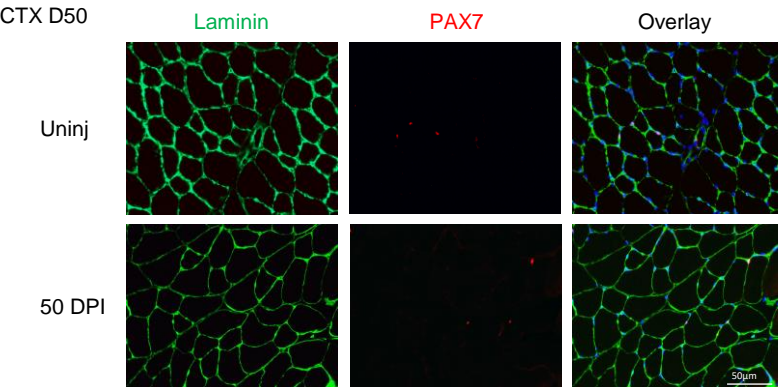

C

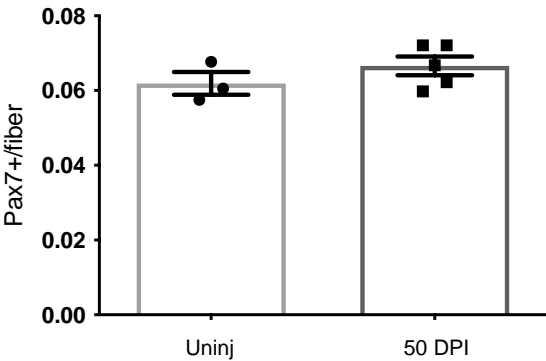

Supplement: Supplementary file 1 — Additional file 1: Fig. S1: A) Representative images of uninjured muscle (uninj) and 50 days post-cardiotoxin injury (50 DPI) stained by hematoxylin and eosin (H&E), showing complete fiber regeneration by 50 days after injury. B) Representative images of Pax7 and laminin staining in uninjured muscle or 50 days after cardiotoxin injection. C) Quantification of the number of Pax7+ cells per muscle fiber as in (B). n = 3 independent samples. Data are shown as mean ± S.E.M. [file 13287_2023_3492_MOESM1_ESM.pdf]

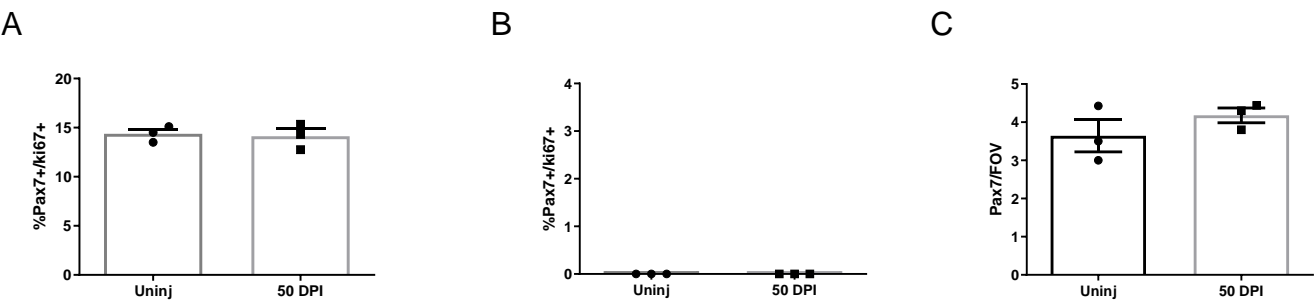

Supplement: Supplementary file 2 — Additional file 2: Fig. S2: A) Quantification of the number of Pax7+ cells per field of view (FOV) after SCs isolation from injured (50 days post-injury (50DPI)) or uninjured muscle assessed by immunofluorescence. B) Quantification of the percentage of Pax7+/ki-67+ SCs isolated 50 DPI or uninjured muscle assessed as in (A). C) Quantification of cell diameter, expressed in microns, of the SCs isolated 50 DPI or uninjured muscle. n = 3 independent samples. Data are shown as mean ± S.E.M. [file 13287_2023_3492_MOESM2_ESM.pdf]

Supplementary Figure 3

A

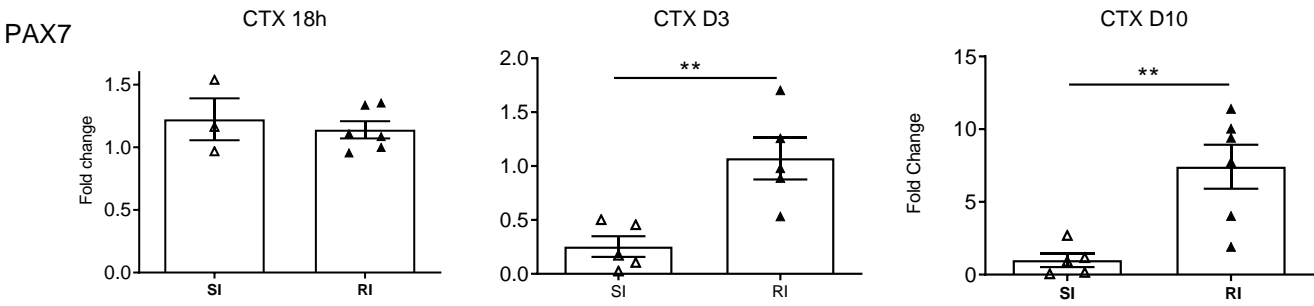

B

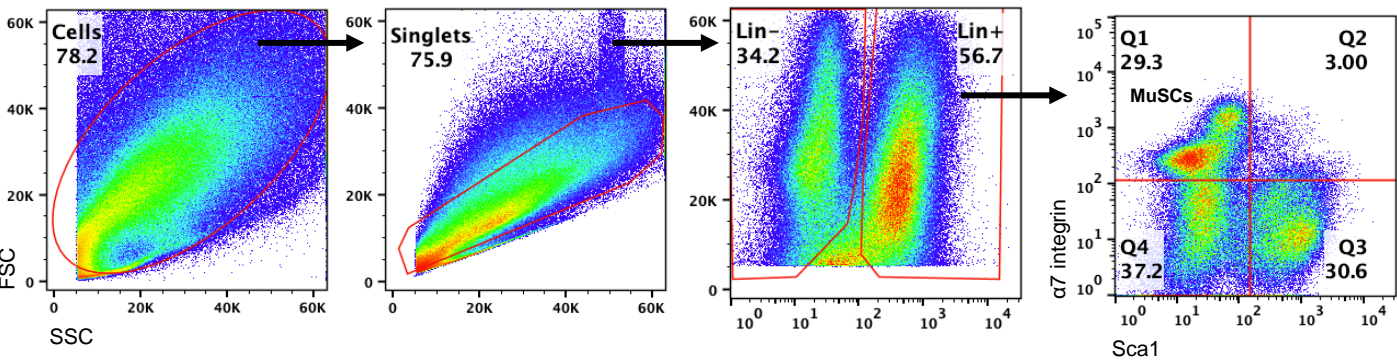

C

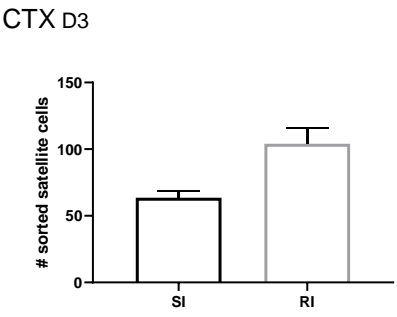

D

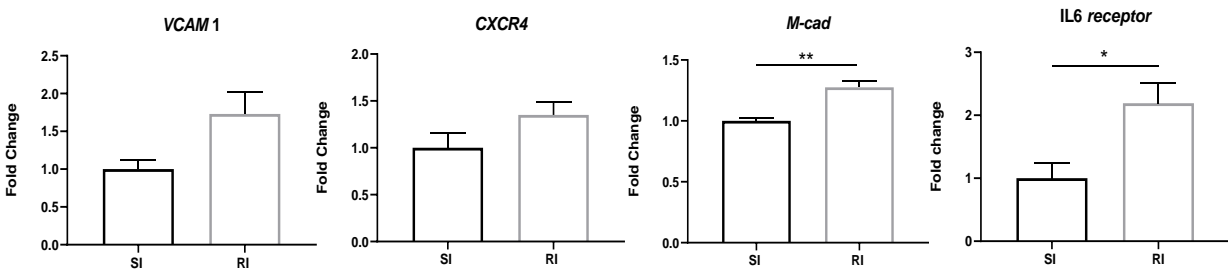

Supplement: Supplementary file 3 — Additional file 3: Fig. S3: A) Quantification of Pax7 relative expression, as measured by RT-qPCR. RNA was extracted from mononuclear cells isolated from muscle 18 h, 3 and 10 days after single or repeated cardiotoxin injury. Data expressed as fold change (2-ddCT) normalized against GAPDH. B) Representative images of the gating strategy for the sorting of SCs, FAPs and macrophages from muscle. C) Total number of SCs sorted from muscle following single or repeated injury. Quantification of VCAM-1, CXCR4, M-cadherin and IL6-r relative expression by RT-qPCR in SCs FACS-sorted from muscle at 3 DPI following single or repeated injury. Data are shown as mean ± S.E.M. * = p < 0.05, * = p < 0.01. [file 13287_2023_3492_MOESM3_ESM.pdf]

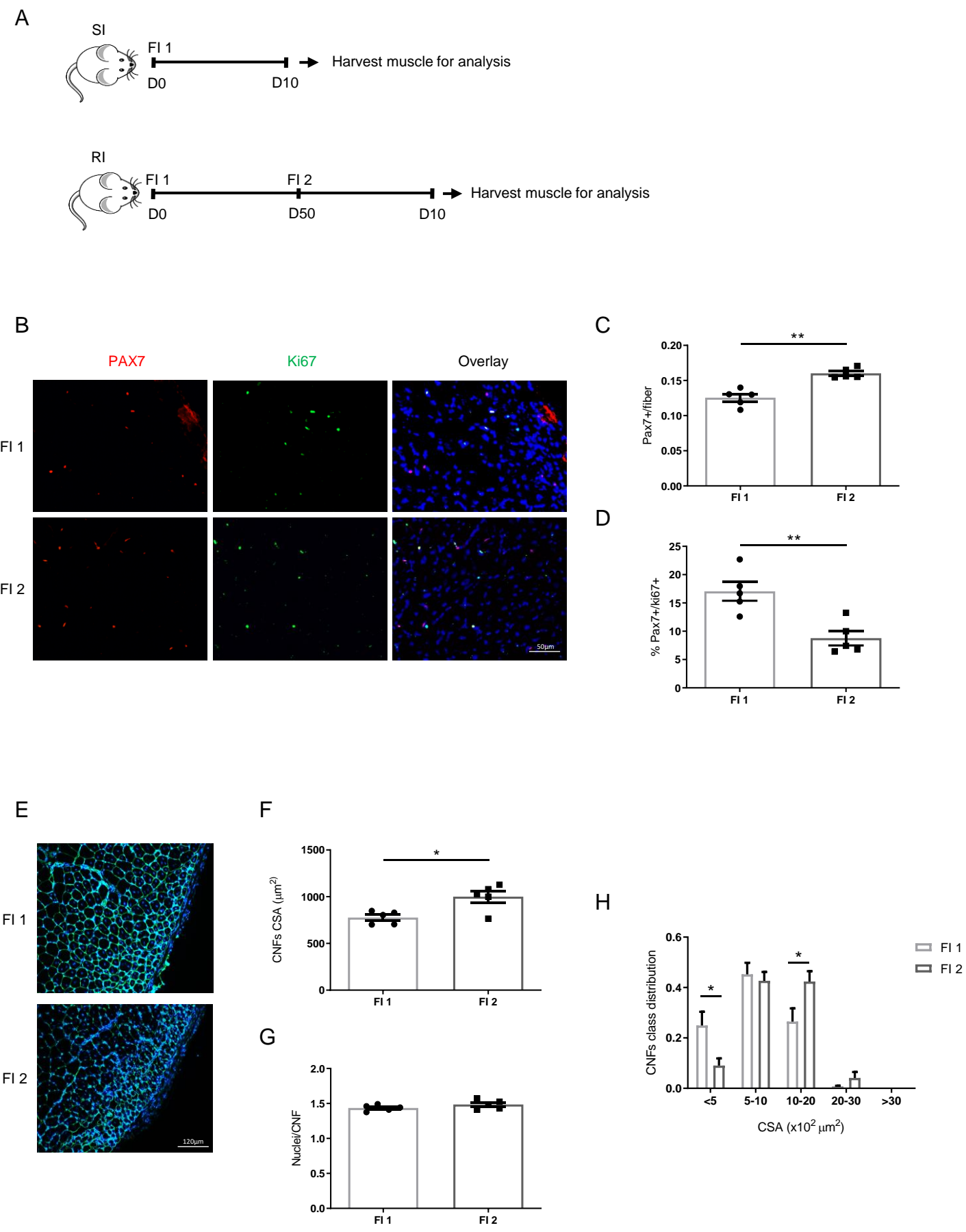

Supplement: Supplementary file 4 — Additional file 4: Fig. S4 A) Schematic diagram of the experimental approach for freeze injury (FI). B) Representative images of Pax7/ki-67 staining of tibialis anterioris muscle sections 10 days after single (FI 1) or repeated freeze injury (FI 2). C-D) Quantification of the number of Pax7+ cells per muscle fiber and quantification of the percentage of Pax7+/ki-67+ SCs in muscle sections, as in (B). E) Representative images of centrally nucleated fibers (CNFs), evidenced by laminin staining, in muscle sections, 10 days after single or repeated freeze injury. F-G) Quantification of the cross-sectional area of the CNFs, expressed in square microns, and quantification of the number of nuclei per CNF, as in (D). F) Quantification of the distribution of CNFs per CSA, expressed in percentage, as in (G). 10 days after single or repeated freeze injury, expressed in percentage. N = 5 independent samples. Data are shown as mean ± S.E.M. * p < 0.05, ** p < 0.01. [file 13287_2023_3492_MOESM4_ESM.pdf]

A

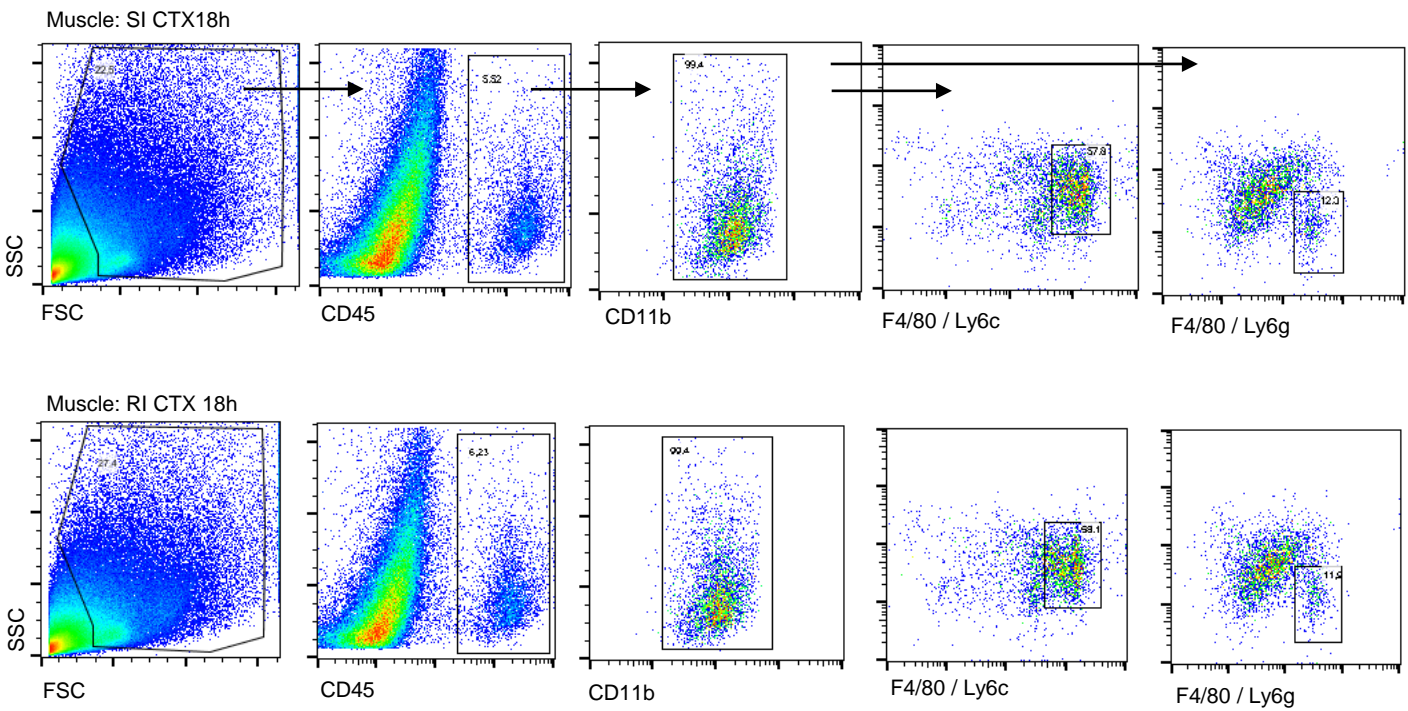

B

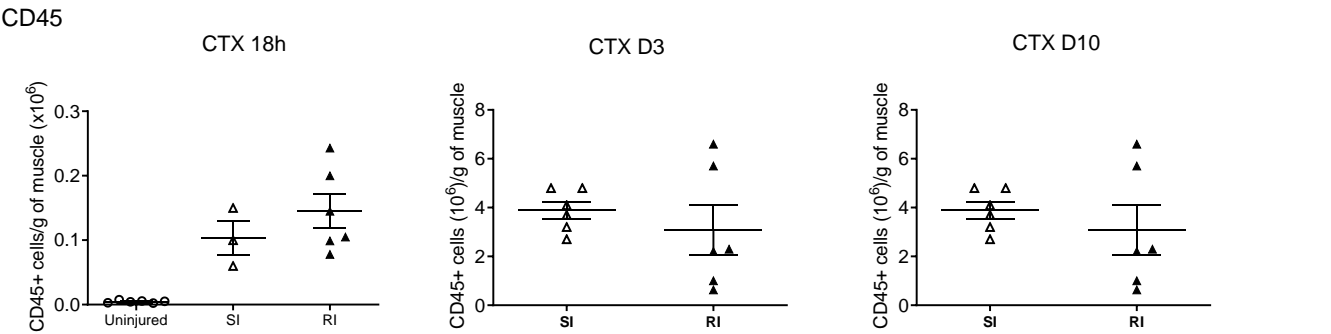

C

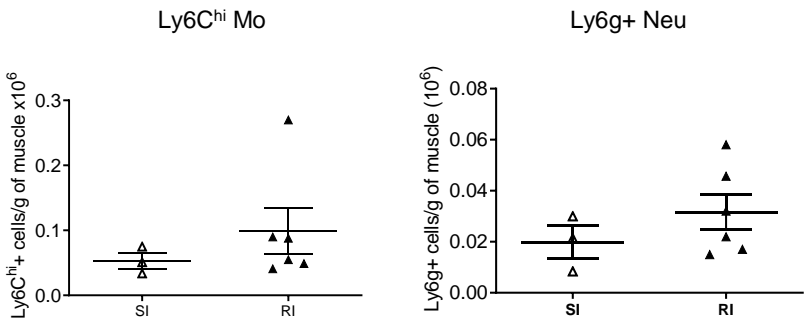

Supplement: Supplementary file 5 — Additional file 5: Fig. S5: A) Representative images of FACS gating strategy for the analysis of immune cells in muscle after single or repeated injury. B) Quantification of the number of hematopoietic (CD45+) cells infiltrating the muscle, 18 h, 3 days and 10 days after cardiotoxin injury, or uninjured muscle, and normalized per gram of tissue. C) Quantification of the number of recently recruited inflammatory monocytes/macrophages (F4/80 + Ly6Chi) and neutrophils (Ly6g+) infiltrating the muscle 3 days after cardiotoxin injury, or uninjured control muscle, normalized per gram of tissue. Data are shown as mean ± S.E.M. [file 13287_2023_3492_MOESM5_ESM.pdf]

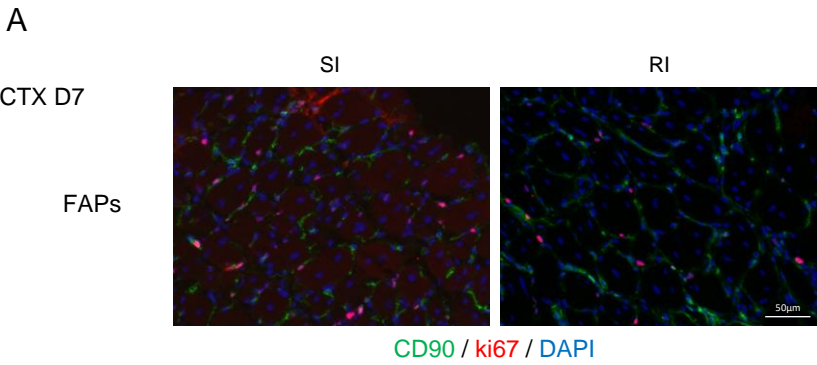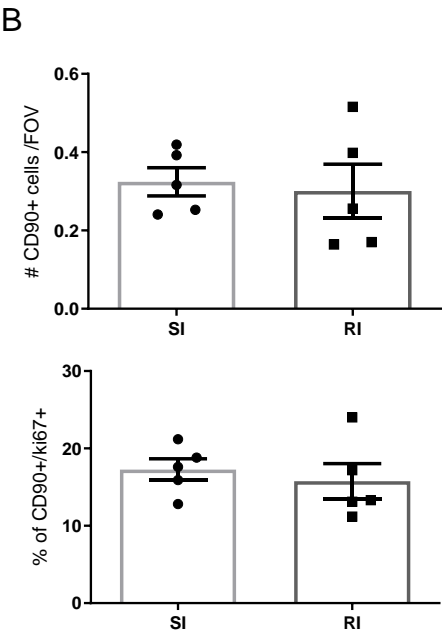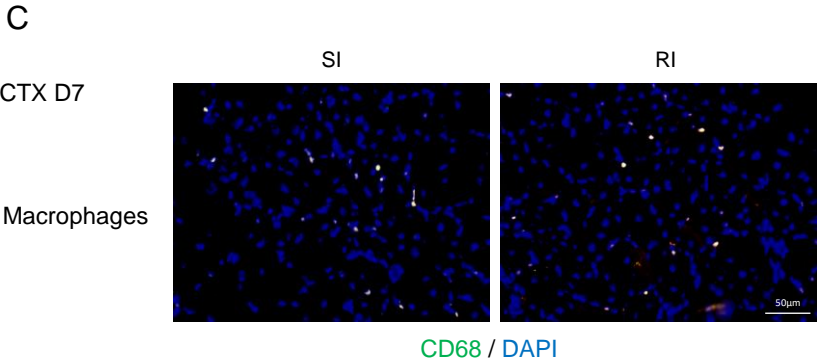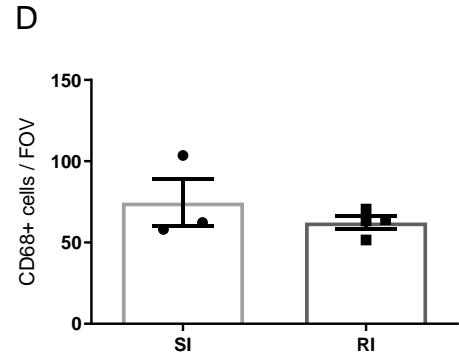

Supplement: Supplementary file 6 — Additional file 6: Fig. S6: A) Representative images of CD90 staining of FAPs in tibialis anterior 7 days after single or repeated injury. B) Quantification of CD90+ cells per FOV, n = 5 independent samples. C) Representative images of CD68+ macrophages staining of tibialis anterior 10 days after single or repeated injury. D) Quantification of the number of macrophages per field of view (FOV). n = 3 (SI) and 4 (RI) independent samples. [file 13287_2023_3492_MOESM6_ESM.pdf]

Supplementary Figure 7

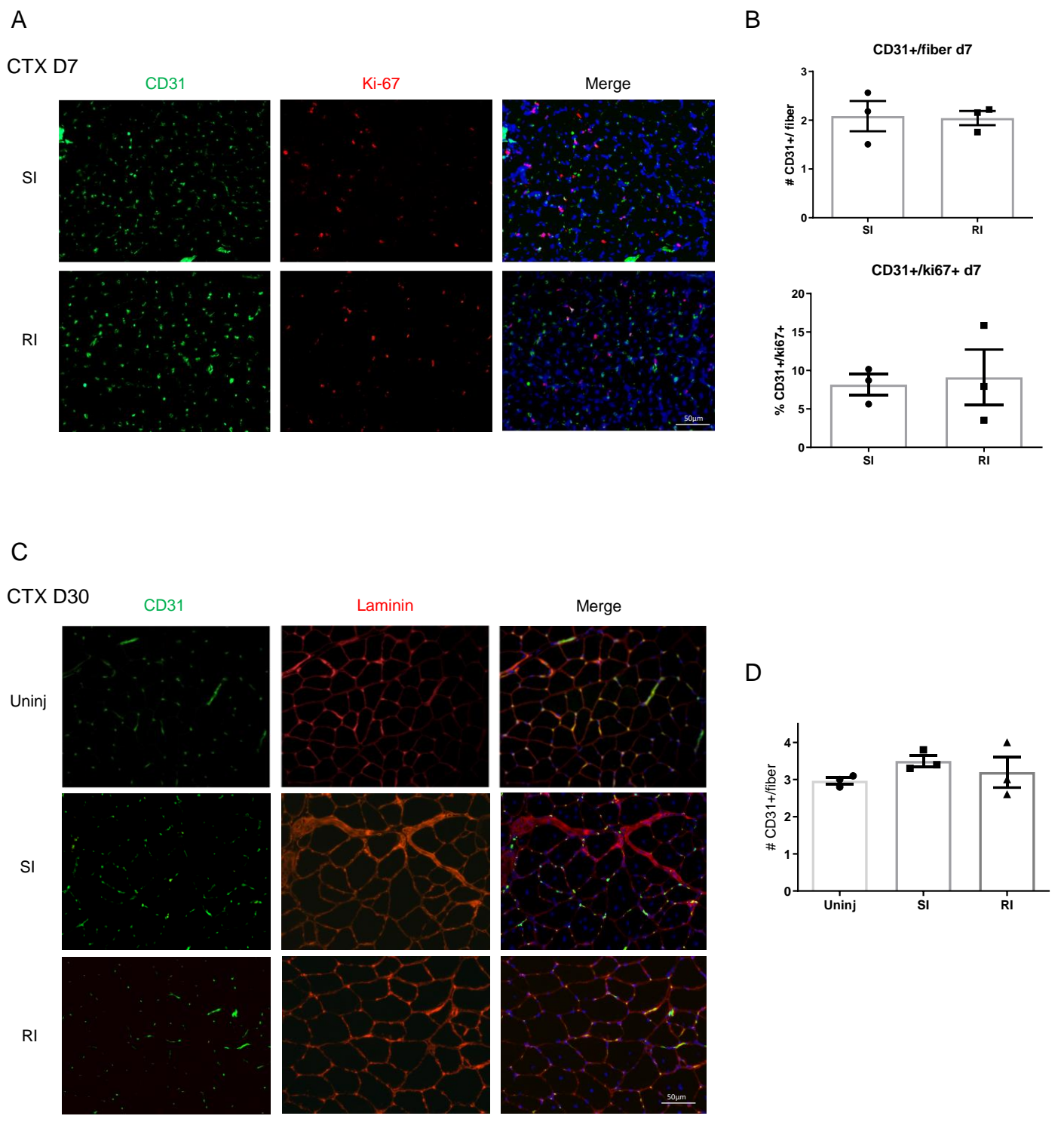

Supplement: Supplementary file 7 — Additional file 7. Fig. S7: A) Representative images of CD31/ki-67 staining of endothelial cells in tibialis anterior muscle sections 7 days after single or repeated injury. B) Quantification of the number of CD31+ cells per fiber and the percentage of CD31+/ki67+ cells in muscle sections, as in (B). C) Representative images of CD31 and laminin staining of tibialis anterior muscle sections 30 days after single or repeated injury. D) Quantification of the number of CD31+ cells per fiber, as in (B). [file 13287_2023_3492_MOESM7_ESM.pdf]
